# Therapeutic Effects of Insulin-Producing Human Umbilical Cord-Derived Mesenchymal Stem Cells in a Type 1 Diabetes Mouse Model

**DOI:** 10.3390/ijms23136877

**Published:** 2022-06-21

**Authors:** Yu Mi Park, Chang Mo Yang, Hee Yeon Cho

**Affiliations:** 1 CHA Advanced Research Institute, 335, Pangyo-ro, Bundang-gu, Seongnam-si 13488, Gyeonggi-do, Korea; 2 Department of Biomedical Science, CHA University, 335, Pangyo-ro, Bundang-gu, Seongnam-si 13488, Gyeonggi-do, Korea; 3 Cell Therapy R&D Center, HansBiomed Corp, 7, Jeongui-ro 8-gil, Songpa-gu, Seoul 05836, Gyeonggi-do, Korea; ycm@hansbiomed.com (C.M.Y.); hycho@hansbiomed.com (H.Y.C.)

**Keywords:** type 1 diabetes, mesenchymal stem cells, insulin-producing cells, transplantation, direct-trans differentiation

## Abstract

In patients with type 1 diabetes (T1D), compromised pancreatic β-cell functions are compensated through daily insulin injections or the transplantation of pancreatic tissue or islet cells. However, both approaches are associated with specific challenges. The transplantation of mesenchymal stem cells (MSCs) represents a potential alternative, as MSCs have tissue-forming capacity and can be isolated from various tissues. The human umbilical cord (hUC) is a good source of freely available MSCs, which can be collected through pain-free, non-invasive methods subject to minimal ethical concerns. We sought to develop a method for the in vitro generation of insulin-producing cells (IPCs) using MSCs. We examined the potential therapeutic uses and efficacy of IPCs generated from hUC-derived MSCs (hUC-IPCs) and human adipose tissue (hAD)-derived MSCs (hAD-IPCs) through in vitro experiments and streptozotocin (STZ)-induced C57BL/6 T1D mouse models. We discovered that compared to hAD-IPCs, hUC-IPCs exhibited a superior insulin secretion capacity. Therefore, hUC-IPCs were selected as candidates for T1D cell therapy in mice. Fasting glucose and intraperitoneal glucose tolerance test levels were lower in hUC-IPC-transplanted mice than in T1D control mice and hAD-IPC-transplanted mice. Our findings support the potential use of MSCs for the treatment of T1D.

## 1. Introduction

Diabetes is classified into two types, namely, type 1 diabetes (T1D) and type 2 diabetes (T2D). Both are chronic diseases that affect the regulation of blood glucose levels. Glucose is the main energy source for most cell types, and insulin is required for cells to uptake glucose. Patients with T1D do not produce insulin, as their pancreatic β-cells are extensively damaged. Pancreatic β-cells are subject to autoimmune attacks, which ultimately leads to their destruction [1]. T1D is largely attributed to genetic causes, although the specific etiology remains elusive [2]. T1D patients exhibit a dysregulated glucose metabolism, experiencing drops of blood glucose below normal levels (acute hypoglycemia). T1D is difficult to manage and greatly limits quality of life.

Patients with T2D are not sensitive to insulin, producing insufficient amounts of the hormone in advanced stages of the disease. Thus, exogenous insulin poses no significant risk to well-being. During T2D development, β-cells secrete a sufficient amount of insulin, which remains unrecognized by cells (insulin resistance), leading to glycemia and subsequent β-cell hypertrophy. T2D can be treated via medication as well as lifestyle adaptations, such as exercise and diet control. Although causes may vary, obesity due to a westernized high-calorie diet is considered the major cause of T2D. Obesity increases the levels of circulating free fatty acids and decreases glucose uptake in muscles, promoting insulin resistance and, subsequently, diabetes [3].

T1D is also known as insulin-dependent diabetes and can occur at any age; however, it is most common among young people [4]. A meta-analysis revealed that T1D has a global incidence of 15 per 100,000 people (95% confidence interval, CI: 0.07–0.12), accounting for approximately 5–10% of all diabetes cases [5,6]. Over time, T1D complications can affect major organs in the body, including the heart, blood vessels, nerves, eyes, and kidneys. Although allogenic pancreatic transplantation and β-cell transplantation are considered highly efficient, they are limited by the low number of donors. Pancreatic transplants are routinely conducted by a small number of institutions in North America, Europe, and Australia. If donors appear in other countries, pancreatic islets must be separated through standardized methodology based on enzymes and mechanical digestion. Given the islet isolation team’s 24-h availability and the complex coordination associated with transplantation, the procedure is performed in a small number of highly specialized facilities. In addition, pancreatic procurement and transportation must minimize cold ischemia time, and the islet facility must be able to transport the islet to the local center for transplantation, further limiting appropriate facilities [7]. As the incidence and prevalence of diabetes are rising worldwide, medical expenses associated with lifelong insulin administration and pancreatic transplantation are also increasing. Therefore, the development of novel effective treatments for T1D is imperative [8].

Stem cells may provide an alternative treatment option for T1D. Among the various types of stem cells, mesenchymal stem cells (MSCs) are considered to be relatively easy and safe to use as autologous or allogenic “raw materials” for clinical trials. Further, they are currently explored as treatment options for diverse diseases. MSCs are found in various tissues, including the umbilical cord, adipose tissue, the brain, the spleen, the pancreas, the liver, the kidney, the lung, and the thymus, thus not being limited to tissues of mesenchymal origin (e.g., bone marrow, fat, muscle, and bone). Although all MSCs share a common and unique phenotype, they also exhibit characteristics dependent on the tissue of origin [9,10]. 

Although MSCs isolated from distinct tissues exhibit similar morphological characteristics and surface antigen expressions, populations differ in terms of differentiation ability, proliferative capacity, and immune-regulatory effects [11,12]. The use of MSCs obtained from the umbilical cord, placenta, and amniotic membrane is not associated with ethical issues; additionally, the method of harvesting MSCs from somatic tissues is not invasive. Moreover, these tissues host various embryonic cells, including MSCs, endothelial stem/progenitor cells, and hematopoietic stem cells, with superior proliferative capacities, longevities, differentiation potentials, and levels of passage compared with adult stem cells [13].

In this study, we sought to develop a novel method that allows the generation of insulin-producing cells (IPCs) from MSCs and their use as cell therapy for T1D treatment. To this end, we compared the characteristic features and insulin secretion capacities of pancreatic β-like cells through the direct trans-differentiation of MSCs derived from the human umbilical cord (hUC) and human adipose tissue (hAD). Furthermore, we induced the differentiation of IPCs and transplanted them into a T1D mouse model to assess their efficacy as a treatment modality.

## 2. Results

### 2.1. Characterization of hUC-MSCs and hAD-MSCs

**Figure 1:**
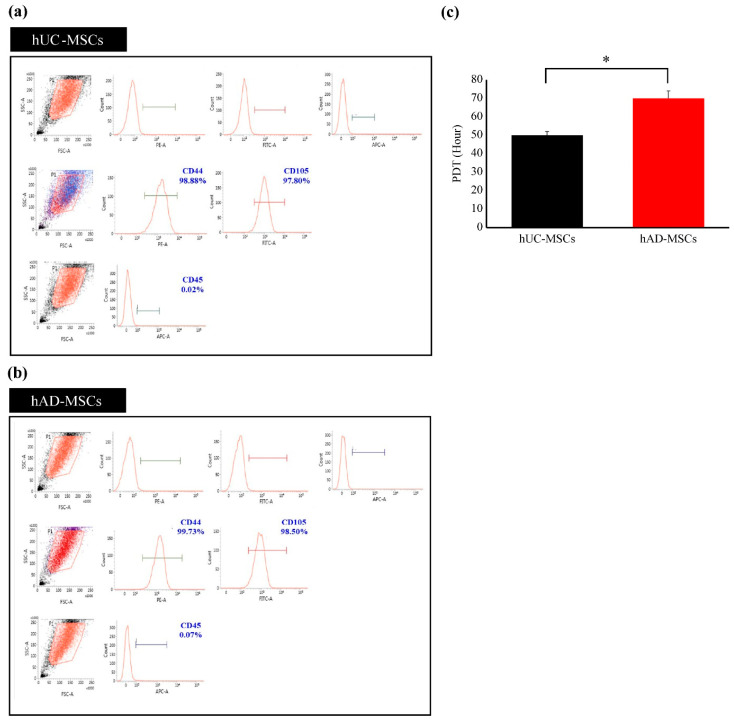
Analysis of mesenchymal stem cell markers using flow cytometry. In a sample of MSCs, ≥97% of MSCs must express CD44 as well as CD105 but not (<1%) CD45. (**a**) Marker expression in hUC-MSCs. (**b**) Marker expression in hAD-MSCs. (**c**) Population doubling time for passages 1–3. The doubling times of the hUC-MSCs and hAD-MSCs were calculated as per the Patterson formula. Data are presented as the mean ± standard error of the mean (*n* = 5; hUC-MSCs vs. hAD-MSCs, * p < 0.05). Abbreviations: hUC-MSCs, mesenchymal stem cells derived from human umbilical cord tissue; hAD-MSCs, mesenchymal stem cells derived from human adipose tissue.

As expected for MSCs, both hUC-MSCs and hAD-MSCs were negative for CD45 and positive for CD44 as well as CD105 (Figure 1a,b) [14]. They exhibited a spindle-shaped and fibroblast-like morphology. Furthermore, hUC-MSCs had a shorter doubling time than hAD-MSCs, dividing more rapidly during passages 1–3 (Figure 1c).

### 2.2. Induction of IPCs from hUC-MSCs and hAD-MSCs

**Figure 2:**
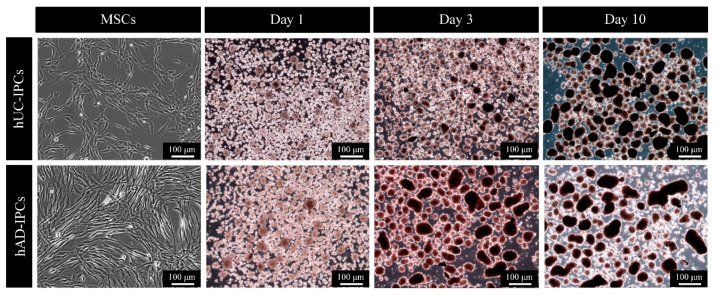
Induction of cultured hUC-MSCs and hAD-MSCs into IPCs using a modified insulin–transferrin–selenium protocol. IPCs differentiation were induced in three steps over 10-day. Each step was marked by a change in medium composition. The differentiation of IPCs from hUC-MSCs (hUC-IPCs) went from step 1 to step 3, as cells gradually attained a round shape. The differentiation of IPCs from hAD-MSCs (hAD-IPCs) resulted in a significantly larger cell size during step 2. In step 3, the cells showed a tendency to adhere together as they grew. Images of all fluorescently stained cells were acquired using a ×100 objective, and representative images are shown. Abbreviations: IPCs, insulin-producing cells; MSCs, mesenchymal stem cells; hUC-MSCs, mesenchymal stem cells derived from human umbilical cord tissue; hAD-MSCs, mesenchymal stem cells derived from human adipose tissue; hUC-IPCs, insulin-producing cells from human umbilical cord-derived mesenchymal stem cells; hAD-IPCs, insulin-producing cells from human adipose-derived mesenchymal stem cells.

First, the intrinsic characteristics of hMSCs were confirmed. Next, we compared the capacity of hUC-MSCs and hAD-MSCs for IPC induction (Figure 2). The IPCs derived from hUC-MSCs had a small diameter and were more stable than those derived from hAD-MSCs, which indicated that the former had a suitable shape and cluster size for transplantation experiments.

IPCs derived from hAD-MSCs (hAD-IPCs) rapidly increased in islet equivalents (IEQs), along with the cluster size (≥500 μm) in induction step 3, when compared to those derived from hUC-MSCs (≤200 μm). However, IPCs derived from hUC-MSCs steadily increased in both IEQ and the number of cluster cells from step 1 to step 3. These were usually distributed as 100–150 μm and 150–200 μm in size during induction step 3. In contrast, the IPCs derived from hAD-MSCs were ≥350 μm during the induction step 3. Furthermore, hAD-IPCs had a cluster cell size >350–500 μm. For cluster cells greater than the normal size, changes in the cell microenvironment, including the intracellular pH level and oxygen membrane transmission, were observed [15]. These changes decreased cellular function and affected the cell viability. In addition, smaller cluster sizes were preferable to enable drawing up clusters with a catheter when IPCs were transplanted into mice with induced T1D. Transplantation also required a suitable cluster size because of their guaranteed cell viability and functional capacity.

### 2.3. Functions and Characteristic Features of Insulin-Producing β-like Cells Derived from hUC-MSCs and hAD-MSCs

**Figure 3:**
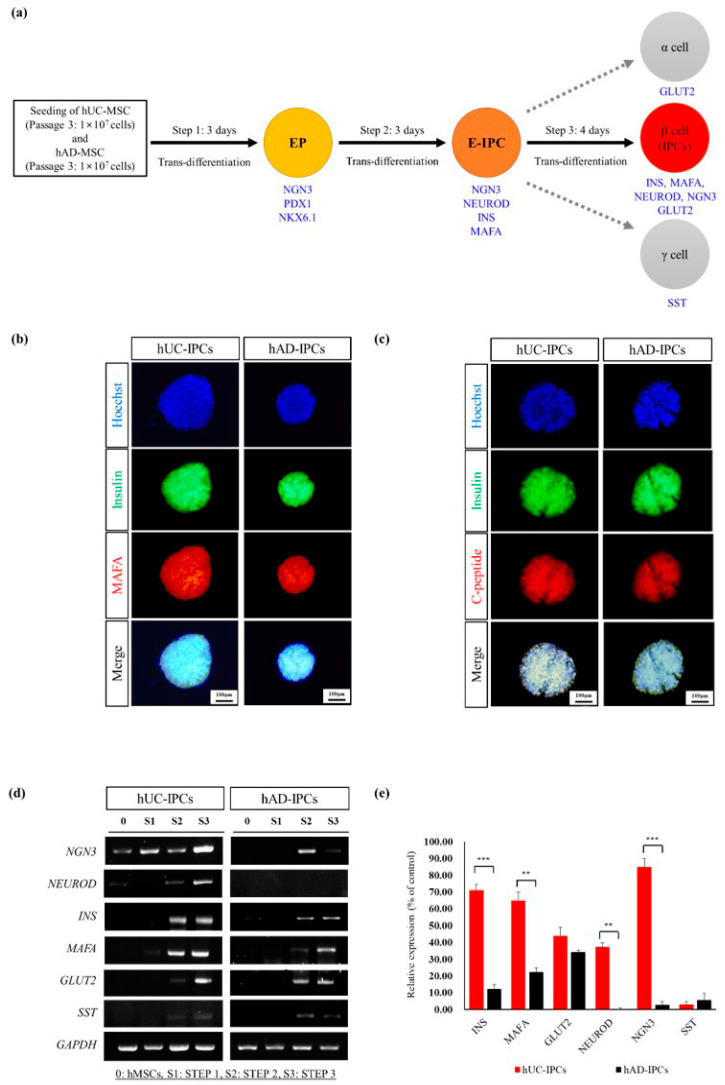
Trans-differentiation of mesenchymal stem cells. (**a**) The trans-differentiation protocol for obtaining pancreatic β-cells. Key transcription factor hierarchy during pancreas development. (**b**) Immunofluorescent detection of MAFA (Red) and insulin (green), as well as 4′,6-diamidino-2-phenylindole (DAPI) nuclear staining (blue) in cultured hUC-MSCs and hAD-MSCs during step 3. The merged image of MAFA and insulin is shown. Images of all fluorescently stained cells were acquired using a ×100 objective, and representative images are shown. (**c**) Immunofluorescent detection of C-peptide (red), insulin (green), as well as DAPI nuclear staining (blue) in cultured hUC-MSCs and hAD-MSCs during step 3. The merged image of C-peptide and insulin-producing β-cells is shown. Images of all fluorescently stained cells were obtained using a ×100 objective, and representative images are shown. Differentiation of hUC-MSCs and hAD-MSCs into IPCs. (**d**) RT-PCR analysis of pancreatic cell marker expression in IPCs differentiated from hUC-MSCs and hAD-MSCs. Marker genes include neurogenin-3 (*NGN3*), neuronal differentiation (*NEUROD*), *INS*, MAF BZIP transcription factor A (*MAFA*), glucose transporter 2 (*GLUT2*), somatostatin (*SST*), normalized to the glyceraldehyde-3-phosphate dehydrogenase (*GAPDH*) gene. (**e**) Quantitative analysis of mRNA expression during IPC differentiation from hUC-IPCs and hAD-IPCs during step 3. Results are presented as the mean ± standard error of the mean. Significant differences between hAD-IPCs and hUC-IPCs are noted at ** *p* < 0.01; *** *p* < 0.001. Abbreviations: E-IPC, early insulin-producing cells; EP, endocrine precursor; hAD-MSCs, mesenchymal stem cells derived from human adipose tissue; hUC-MSCs, mesenchymal stem cells derived from human umbilical cord tissue; IPC, insulin-producing cells; hUC-IPCs, insulin-producing cells from human umbilical cord-derived mesenchymal stem cells; hAD-IPCs, insulin-producing cells from human adipose-derived mesenchymal stem cells; 0, before differentiation; S1, step 1; S2, step 2; S3, step 3; GLUT2, glucose transporter 2; NEUROD, neurogenic differentiation; NGN3, neurogenin 3; GAPDH, glyceraldehyde 3-phosphate dehydrogenase.

**Figure 4:**
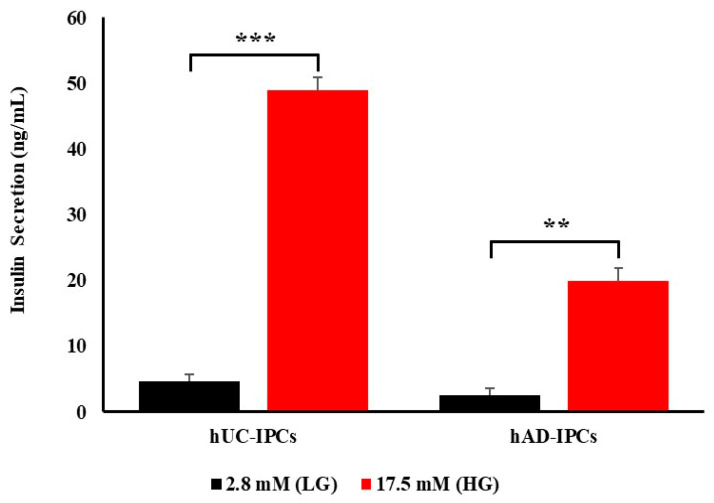
Insulin secretion from pancreatic islets in hUC-IPCs and hAD-IPCs, as evaluated using the GSIS test. Low- (2.8 mM) and high-glucose (17.5 mM) conditions were used to stimulate hUC-IPCs and hAD-IPCs. Insulin secretion was quantitatively measured using insulin enzyme-linked immunosorbent assay (ELISA). Data are presented as the mean ± standard error of mean. (*n* = 3 per IPC type; hUC-MSCs vs. hAD-MSCs, ** p < 0.01; *** *p* < 0.005). Abbreviations: LG, low glucose; HG, High glucose; GSIS, glucose-stimulated insulin secretion; hUC-IPCs, insulin-producing cells from human umbilical cord-derived mesenchymal stem cells; hAD-IPCs, insulin-producing cells from human adipose-derived mesenchymal stem cells.

The pancreatic transcription factor hierarchy changes during pancreatic development, as numerous transcription factors are involved in the differentiation of insulin-producing β-like cells (Figure 3a). To confirm the characteristic features of IPCs derived from hUC-MSCs and hAD-MSCs, the protein levels of insulin, MAFA, and C-peptide were measured using immunocytochemistry. All three were upregulated upon the trans-differentiation of hUC-MSCs into IPCs, to a greater extent than those observed in hAD-MSCs differentiated into IPCs (Figure 3b and Figure 4). Moreover, IPCs expressed proteins similar to insulin-producing β-like cells and were well established.

The expression levels of pancreatic genes in hUC-MSCs and hAD-MSCs during IPC differentiation steps 1–3 were measured using reverse transcriptase-polymerase chain reaction (RT-PCR) and compared (Figure 3d). *INS*, *MAFA*, *GLUT2*, *NEUROD*, and *NGN3* expression levels were higher in hUC-IPCs than in hAD-IPCs at IPC differentiation step 3. In particular, all the above-mentioned genes exhibited a significantly higher expression in hUC-IPCs than in hAD-IPCs. The transcription factor MAFA is a β-cell-specific activator, which is associated with insulin regulation and is regulated by blood glucose levels as well as oxidative stress [15,16]. In addition, GLUT2, a cellular membrane transporter that transfers glucose between the liver and blood, was highly expressed in hUC-IPCs during IPC differentiation steps 2 and 3. GLUT2 expression rapidly increased in hUC-IPCs during IPC differentiation step 3 when compared to step 2. Somatostatin was expressed at lower levels in hUC-IPCs than in hAD-IPCs during step 3 of differentiation.

Next, the insulin secretion capacity of hUC-IPCs and hAD-IPCs was examined using a glucose-stimulated insulin secretion (GSIS) assay. Both cell types secreted insulin in response to glucose stimulation, with hUC-IPCs secreting much higher levels of insulin than hAD-IPCs at low glucose (2.8 mM) and high glucose (17.5 mM) levels (Figure 4). 

Based on these results, hUC-IPCs were selected for use in the mouse T1D model, owing to the similar morphology to stable insulin-producing β-like cluster cells, the smaller diameter of cluster cells, and the higher insulin secretion capacity compared to hAD-IPCs. The hUC-IPCs also exhibited higher mRNA expression levels of *INS*, *MAFA*, *NEUROD*, *GLUT2*, and *NGN3* than hAD-IPCs.

### 2.4. Therapeutic Effects of IPCs in the T1D Animal Model

**Figure 5:**
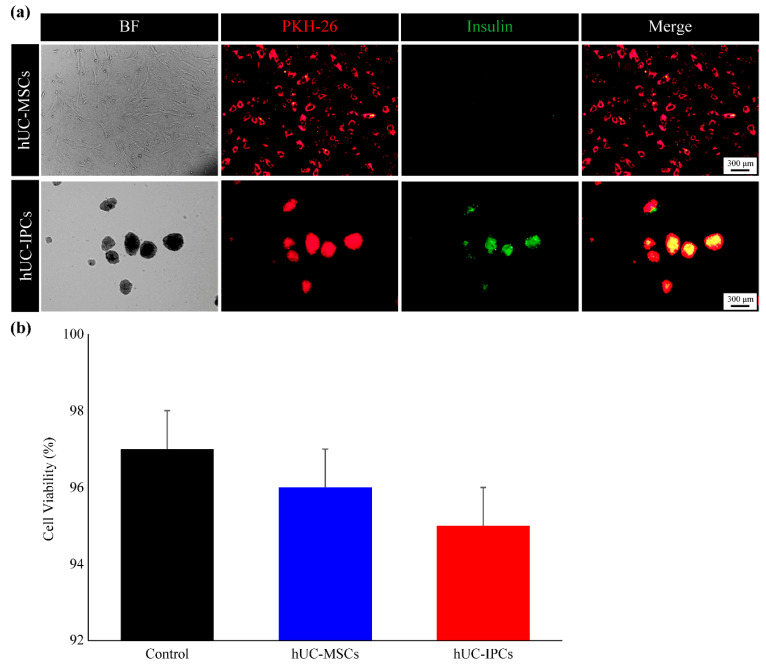
Identification of PKH-26-Red-stained hUC-MSC-derived trans-differentiated IPCs. (**a**) All fluorescent stained cells were observed under ×40 objectives, and representative images are shown. (**b**) Cell viability in controls, hUC-MSCs, and hUC-IPCs labeled with PKH26-Red determined using MTT assays. Unlabeled hUC-MSCs were used as controls. Data are presented as the mean ± standard error of the mean. (*n* = 3 per cell type). Abbreviations: BF; bright field, hUC-IPCs, insulin-producing cells from human umbilical cord-derived mesenchymal stem cells; hUC-MSCs, mesenchymal stem cells derived from human umbilical cord tissue; IPCs, insulin-producing cells; MTT (3-(4,5-dimethylthiazol-2-yl)-2,5-diphenyltetrazolium bromide); hUC-MSCs, mesenchymal stem cells derived from human umbilical cord tissue; hUC-IPCs, insulin-producing cells from human umbilical cord-derived mesenchymal stem cells.

We examined whether hUC-IPC transplantation into a mouse T1D model would improve blood glucose levels. The hUC-MSCs stained with PKH26-Red maintained PKH26-Red expression, co-localizing with insulin staining in vitro (Figure 5a). In addition, the cell viability of hUC-MSCs and hUC-IPCs labeled with PKH26-Red was assessed using MTT proliferation assays. When compared with the controls (hUC-MSCs without PKH26-Red dye), hUC-MSCs and hUC-IPCs stained with PKH26-Red showed no difference in survival before transplantation (Figure 5b). Therefore, PKH26-Red-stained cells were selected for in vivo transplantation experiments.

**Figure 6:**
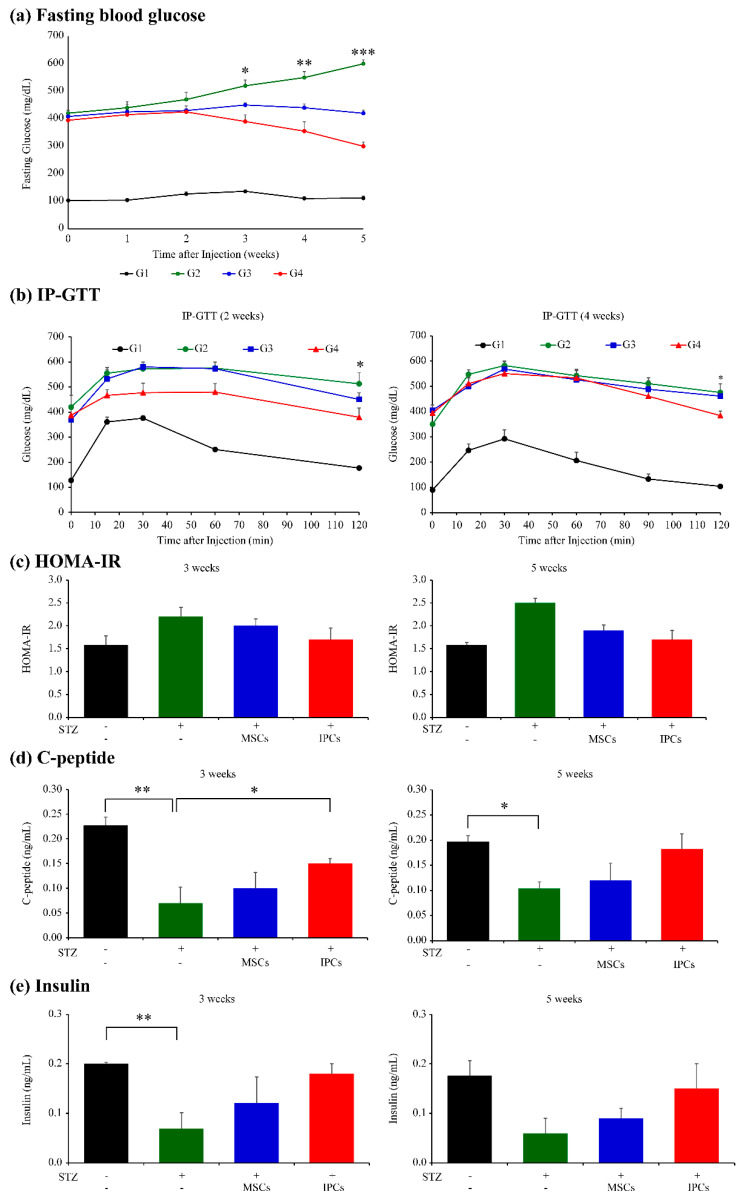
Functional analysis after hUC-MSC and hUC-IPC transplantation in vivo. (**a**) Blood glucose levels after hUC-MSCs and hUC-IPCs transplantation. Glucose levels were monitored using tail-vein blood samples. Results are presented as the mean ± standard error (*n* = 10 per group; G2 vs. G4, ** *p* < 0.001; *** *p* < 0.005). (**b**) T1D mice received hUC-MSCs and hUC-IPCs. Mice that responded to the transplantation and achieved normoglycemia were assessed at the end of 5 weeks for insulin sensitivity, as measured using IP-GTT. Results are presented as the mean ± standard error of the mean (*n* = 5 per group; significant differences are noted between G2 and G4 at * p < 0.05). (**c**) The homeostasis model assessment (HOMA) insulin resistance (IR) index at 3 and 5 weeks after cell transplantation (*n* = 5 per group). Data are presented as the mean ± standard error. (**d**) Mean C-peptide level in T1D model mice and T1D model mice receiving cell therapy, at 3 and 5 weeks following cell transplantation (*n* = 5 per group). Data are presented as the mean ± standard error (G1 vs. G2, * p < 0.05; G2 vs. G4, ** *p* < 0.001). (**e**) Serum insulin levels after transplantation of hUC-MSCs and hUC-IPCs measured using insulin enzyme-linked immunosorbent assay (ELISA) at 3 and 5 weeks. Data are presented as the mean ± standard error of mean (*n* = 5 per group; G1 vs. G2, ** *p* < 0.001). Abbreviations: G, group; hUC-IPCs, insulin-producing cells from human umbilical cord-derived mesenchymal stem cells; hAD-IPCs, insulin-producing cells from human adipose tissue-derived mesenchymal stem cells; T1D, type 1 diabetes; IP-GTT, intraperitoneal glucose tolerance test; STZ, streptozotocin; MSC, mesenchymal stem cell; IPCs, insulin-producing cells; HOMA-IR, homeostasis model assessment for insulin resistance; ELISA, enzyme-linked immunosorbent assay.

The therapeutic efficacy of transplanted cells was assessed using group (G) 1 and G2 mouse models as controls for the G3 and G4 models. Fasting blood glucose (FBG) levels significantly increased to 300 mg/dL in the T1D mouse model (G2) compared with those in normal controls (G1), confirming the T1D model establishment. Further, compared with G2 mice, T1D mice receiving hUC-IPCs (G4) showed decreased FBG levels, with FBG levels significantly decreasing at 3, 4, and 5 weeks following transplantation (Figure 6a). Next, an intraperitoneal glucose tolerance test (IP-GTT) was performed at 2 and 4 weeks (Figure 6b). All groups demonstrated increased blood glucose levels for up to 30 min after glucose administration, and a subsequent decrease for up to 120 min at 2 and 4 weeks. Among the experimental groups, blood glucose levels significantly reduced at 120 min in G4 mice when compared with those in G2 mice. The homeostasis model assessment (HOMA) values for insulin resistance (IR) was lower in G4 mice than in G2 and G3 mice. In addition, the HOMA-IR values of G1 and G4 mice were approximately the same at 2 and 4 weeks (Figure 6c).

C-peptide levels decreased significantly in G2 compared with those in the control group. In addition, the amount of C-peptide was significantly higher in G4 than in G2 at 3 weeks following transplantation (Figure 6d). The C-peptide concentration in G4 was approximately equivalent to that observed in G1 at 5 weeks following transplantation. Meanwhile, the C-peptide concentration measured in G3 at 3 weeks after transplantation was not different from that measured at 5 weeks after transplantation. Similarly, the blood insulin concentration was determined at 3 and 5 weeks after transplantation (Figure 6e). Insulin concentration was shown to significantly decrease in G2 compared with that in G1. Further, the insulin concentration measured at 3 weeks indicated that insulin levels were higher in G4 than in G2 and G1. Collectively, these results confirmed that hUC-IPC transplantation can help reduce blood glucose levels.

**Figure 7:**
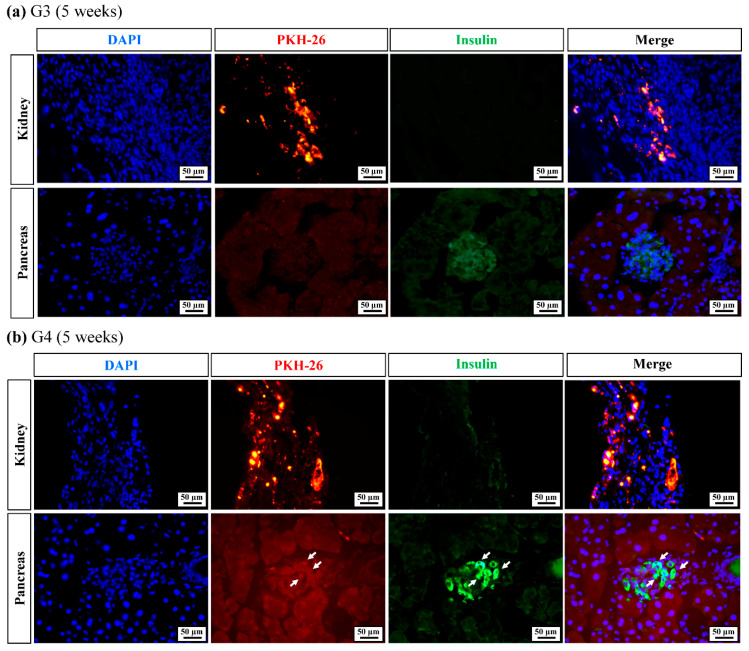
Application of cell therapy agents in a T1D mouse model. All fluorescently stained cells were observed under a (**a**) ×40 objective and a (**b**) ×50 objective, and representative images are shown. The white arrows indicate merged cells. Abbreviations: G, group; DAPI, 4′,6-diamidino-2-phenylindole.

The kidneys and pancreases of G3 and G4 mice were isolated after euthanasia at 5 weeks post-transplantation. PKH26-Red-stained hUC-MSCs and hUC-IPCs were identified using immunofluorescence staining (Figure 7). In G3 mice, hUC-MSCs were present in the kidney where transplantation was performed, and no proliferation was observed in the pancreas. Low levels of insulin staining were observed in the pancreas after 5 weeks (Figure 7a). PKH26-Red was also detected in the kidneys of G4 mice, and insulin levels were higher in the pancreas of G4 mice than in those observed of G3 mice at 5 weeks. Furthermore, insulin co-localized with PKH26-Red-stained hUC-IPCs in the pancreas (white arrow in Figure 7b). 

Therefore, we concluded that the transplanted hUC-MSCs and hUC-IPCs were maintained within recipient mice for up to 5 weeks. More importantly, the transplanted hUC-IPCs migrated to the pancreas where they co-localized with insulin expression. Lastly, transplanted hUC-IPCs increased insulin secretion, with insulin in turn regulating blood glucose levels to a greater extent than that observed in G2 and G3 mice.

## 3. Discussion

T1D is an autoimmune disease characterized by the destruction of pancreatic β-cells by autologous T cells. So far, no clear cause has been identified, and no effective treatment is widely available. As in patients with T2D, the blood glucose levels in T1D patients are controlled through injections of exogenous insulin. Pancreatic transplantation remains the only available and effective cure for T1D. As per the Edmonton protocol, islet cells can be separated from the pancreas of a donor and transplanted into T1D patients. After transplantation of a contributor-guided islet, blood glucose regulation can be re-established up to half of baseline levels. However, islet cell transplantation is limited by a shortage of donors, the need for the use of post-transplant immunosuppressive drugs (ISDs), and the use of wrong injections. To overcome these complications, in vivo experiments in diabetic animals using insulin-like cells derived from hMSCs, human-induced pluripotent stem cells, and human embryonic stem cells have been conducted, with only a few reporting long-lived efficacy [17,18,19,20]. The goal of T1D therapy is restoring the impaired pancreatic β-cell function. Currently, hMSCs are the most accessible type of therapeutic stem cells and thus are used in various studies for their characteristic functions. If hMSC-based insulin-producing β-like cells are successfully established, stem cell transplantation represents an ideal approach to restore insulin production within the pancreas [21]. Although an optimal treatment for acute or chronic graft-versus-host disease (GvHD) has not yet been developed, hMSCs have significant therapeutic potential. Since hMSCs promote immunosuppressive and immunomodulatory environments through a number of mechanisms, including protein/peptide/hormone secretion, mitochondrial delivery, and the transfer of exosomes or micelles containing RNA and other molecules, hMSCs are also considered a promising medium for cell therapy against T1D [22,23,24,25,26]. Although pancreatic insulin-producing β-like cells have been generated from MSCs in vitro and transplanted in diabetic animal models, their underlying mechanisms of action have not yet been elucidated [23]. MSCs express chemokine receptors and play an important role in the homing effect [27] as well as in pancreatic regeneration [23,24]. Furthermore, the systemic administration of MSCs in STZ-induced diabetic rats resulted in an increase in β-cell volume [24,28]. 

MSCs have great potential as mediator cells in regenerative medicine and tissue engineering due to their great proliferative capacity, the ability to differentiate into multiple cell lineages, easy separation from various tissues, low immunogenicity, and paracrine activity [29,30]. Stem cells promote skin homeostasis and wound regeneration, maintaining epidermal cells between pores and hair follicles through various genetic and posterior signaling pathways [31,32,33]. It has been previously shown that stem cell-secreted growth factors and cytokines promote tissue regeneration and recovery [34,35]. Although bone marrow derived MSCs are the most extensively studied, MSCs have also been isolated from blood, peripheral blood, adipose tissue, umbilical cord, amniotic membrane, and dermal tissue [36,37]. MSCs have been shown to resolve hyperglycemia in a T1D animal model, as they enhance insulin secretion and pancreatic regeneration [38,39,40,41,42]. However, MSCs from distinct sources may have different biological characteristics and functional capacities as cell therapy products for T1D. 

In this study, we sought to verify the efficacy of hUC-MSCs and hAD-MSCs for the generation of pancreatic insulin-producing β-like cells and identify candidate IPCs for T1D cell therapy. Based on our findings, we attempted to induce the differentiation of human IPCs using hUC-MSCs to develop a therapeutic agent for T1D.

First, we discovered that hUC-MSCs and hAD-MSCs exhibit growth abilities, surface marker expression, and IPC differentiation capacity, even though they differ in tissue origin. In addition, they were morphologically similar, with a spindle shape and helical growth, when fostered under the same conditions. However, the proliferation doubling time (PDT) of hUC-MSCs was faster than that of hAD-MSCs, and the IPC colony formation rate of hUC-MSCs was higher than that of hAD-MSCs at the same passage. Although these variables may be affected by the donors, it is likely that these differences are attributable to the source organ [43]. 

We further compared functional differences by direct trans-differentiation of hAD-MSCs and hUC-MSCs into IPCs. Our results revealed that hUC-MSCs ranged 101–150 μm throughout differentiation steps 1 to 3. Meanwhile, for hAD-MSCs, significantly larger cells (>500 μm) differentiated during step 3 when compared to cells at steps 1 and 2. The size distribution of native pancreatic islets ranges from 50 to 500 μm [44,45]. Numerous studies have suggested that there is a close connection between islet size and function. In general, originally small islets exhibit a greater insulin secretion and cell survival, both in vitro and in vivo [44,45,46,47,48]. Smaller islets secrete more insulin in vitro, being highly effective in achieving normoglycemia when transplanted into diabetic animals [47]. We examined the size effects of direct trans-differentiated IPCs from hUC-MSCs and hAD-MSCs on cell viability, the arrangement of β-like IPCs, and insulin secretion ability in vitro and in vivo. To confirm that the IPCs differentiated from both MSC types were pancreatic β-like cells, we selected similar cell sizes and used immunostaining to assess insulin, MAFA, and C-peptide protein expression. We further analyzed the gene expression levels of *NGN3*, *NEUROD*, *INS*, *MAFA*, *GLUT2*, and *SST* using RT-PCR. In the case of hAD-IPCs, all the above-mentioned genes were expressed in steps 2 and 3 of differentiation. Further, hUC-IPCs exhibited relatively high expressions, especially for insulin. This indicates that hUC-IPCs exhibit favorable morphological and functional IPC characteristics.

Further, we examined the differences in cell size as well as marker expression between IPCs derived from the two different MSC sources. The hUC-IPCs exhibited superior insulin secretion capacities. Additionally, smaller-sized hUC-IPCs exhibited a greater viability, insulin secretion, pancreatic marker expression, and functional regeneration in T1D mice, when compared to hAD-IPCs. These findings indicate that hAD-MSCs attain a larger size at the differentiation onset, while hUC-MSCs maintained a stable morphology and size through trans-differentiation. Therefore, hUC-MSCs are a more promising stem cell source for the treatment of T1D [49]. 

C-peptide is a widely used marker of pancreatic β-cell function. It is secreted by islet β-cells and shares a common precursor with insulin, namely, proinsulin [49,50]. A higher C-peptide concentration was maintained in the blood of G4 animals than that observed in G2 and G3 mice at 3 and 5 weeks following transplantation. Treatment with C-peptide was previously reported to improve neuronal and renal function [49,50,51]. Its therapeutic potential has also been explored against diabetic neuropathy [52,53]. Since C-peptide is secreted in equimolar amounts to insulin, its levels are considered equivalent to those of insulin [54]. Our results showed that the expression patterns of C-peptide and insulin were consistent. Usually, T1D model mice produce little to no insulin and C-peptide [55]. We observed a functional recovery of T1D mice following hUC-IPC transplantation, as determined based on insulin and C-peptide secretion; however, it is possible that hUC-IPCs promote insulin production via islets in the presence of MSCs or IPCs.

Furthermore, hUC-MSCs stained with PKH26-Red and subsequent differentiated IPCs were transplanted into the kidney capsule of mice, whereafter the kidney and pancreas were subjected to immunofluorescence staining. In G3 and G4 mice hUCs were homed within the kidney 5 weeks after transplantation, and weak insulin expression was observed in G3 animals following the immunofluorescence analysis. However, insulin expression in G4 was strongly localized to the pancreas, co-localizing with PKH26-Red stain at 5 weeks post-transplantation.

In summary, we confirmed that IPCs trans-differentiated from hUC-MSCs secreted as much insulin as pancreatic-restored β-cells in recipient mice, drastically lowering blood glucose levels compared to both control T1D mice and T1D mice transplanted with hUC-MSCs. In addition, we observed significant differences between groups (G2 to G4) after 5 weeks, without using ISDs.

It is common to prescribe ISDs to organ transplant recipients in order to prevent the rejection of newly transplanted organs by the immune system. An important aspect of immunosuppressant treatment is patient conformance, as low compliance leads to transplantation failure and eventual re-transplantation or death. To prevent rejection, ISDs should be taken by the recipient for the rest of their life. However, their long-term use is associated with various side effects, such as kidney toxicity, elevated liver enzymes, high blood pressure, and skin cancer. Therefore, some transplant patients prefer not to take immunosuppressive medication. In this study, no death or decline in efficacy were reported in the transplant group, although no ISDs were administered. Further research on their effect within the context of MSCs transplantation in T1D models is necessary, considering that these drugs are suggested to have negative effects on β-cells proliferation [56,57]. If ISDs are not required following MSC transplantation, the associated medical care costs would be reduced while improving the patient quality of life [58]. It should also be noted that the development of IPC cell therapy requires the encapsulation of islet cells and nanotechnology that can safely deliver them and protect them against immune cells [59,60,61,62].

This study did not elucidate the mechanism underlying pancreatic islet regeneration, which should be addressed in future studies. Previous in vitro and in vivo experiments have shown that pancreatic β-like IPCs are effective for the treatment of T1D [63]. Our findings provide a basis for the further development of T1D cell therapy.

## 4. Materials and Methods

### 4.1. Tissues

The hUC tissue samples used in this study were supplied by a maternity hospital in the South Korea. The samples were obtained from five women in the late stage of pregnancy and evaluated separately. The patients had provided informed consent. Ethical approval was obtained from the Public Institutional Review Board (IRB) of the Ministry of Health and Welfare (approval number: P01-201812-33-001).

### 4.2. Isolation and Culture of hUC-Derived MSCs 

Mesenchymal stem cells derived from human umbilical cord tissue (hUC-MSCs) were isolated from the gelatinous tissue of Wharton’s jelly, as previously described [64]. Briefly, the hUC was washed with a 10% antibiotic solution (Sigma-Aldrich, St. Louis, MO, USA) containing Hanks’ Balanced Salt Solution (HBSS; Thermo Fisher Scientific, Waltham, MA, USA) and then cut into 0.3–0.5 cm pieces. The hUC-MSCs were cultured in a mixture of Ham’s F-12K (Kaighn’s) medium (Gibco Invitrogen Life Technologies, Grand Island, NY, USA), Dulbecco’s Modified Eagle Medium high-glucose (DMEM; Gibco Thermo Fisher Scientific, Waltham, MA, USA), and GlutaMAX (Gibco Life Technologies, Carlsbad, CA, USA), supplemented with 10% fetal bovine serum (FBS; Gibco by Life Technologies, Carlsbad, CA, USA) and 1% Penicillin-Streptomycin (Gibco by Life Technologies, Carlsbad, CA, USA) at 37 °C in an incubator with a humidified atmosphere and 5% CO_2_. The medium was refreshed every 48–72 h. Sub-cultured cells were grown in a T-175 flask (Nunc, Naperville, IL, USA) to an 80–90% confluence. Cells at the third passage were used for experiments.

### 4.3. Culture of Human Adipose-Derived MSCs (hAD-MSCs)

The hAD-MSCs were purchased from Lonza (PT-5006, Lonza, Walkersville, MD, USA) and maintained in DMEM, which was supplemented with FBS at 37 °C in a 5% CO_2_ incubator until 80–90% cell confluence. The hAD-MSCs were used during their third passage.

### 4.4. Population Doubling Time 

To confirm MSC proliferation rates, the following formula was used to calculate cell PDT:

PDT = (t × log 2)/ (log Nt − log N0)

where “t” is the cell culture period in h, “Nt” is the number of cells after culture, and “N0” is the number of cells initially inoculated [65].

### 4.5. Characterization of hUC-MSCs and hAD-MSCs

The hUC-MSCs were analyzed using fluorescence-activated cell sorting (FACS) (FACSVerse, BD Bioscience, San Jose, CA, USA) for cell surface marker expression. Cells were harvested using 0.25% Trypsin (Thermo Fisher Scientific, Waltham, MA, USA). For FACS, the sheep anti-human α-CD44 antibody conjugated with PE (R&D Systems, Minneapolis, MN, USA), rat anti-human α-CD105 antibody conjugated with FITC (Abcam, Cambridge, MA, USA), and rat anti-human α-CD45 antibody conjugated with APC (R&D Systems, Minneapolis, MN, USA) were used.

### 4.6. Direct Trans-Differentiation of hUC-MSCs and hAD-MSCs into IPCs

IPCs were induced using a modified three-step, 10-day version of the insulin-transferrin-selenium (ITS) induction method first established in tonsil MSCs [60,66]. In step 1, hUC-MSCs and hAD-MSCs in high-glucose alpha-minimal essential medium (α-MEM; HyClone Laboratories, Logan, UT, USA) were seeded in an uncoated 90 mm × 15 mm dish (Nunc, Naperville, IL, USA) with bovine serum albumin (BSA; Sigma-Aldrich, St. Louis, MO, USA) without 1% fatty acids (Sigma-Aldrich, St. Louis, MO, USA), and ITS media supplement was added at 37 °C in a 5% CO_2_ incubator for 2-day. In step 2, 0.3 mM taurine (Sigma-Aldrich, St. Louis, MO, USA) and 10 mM nicotinamide (Sigma-Aldrich, St. Louis, MO, USA) were added to the differentiation medium detailed in step 1, and cells were incubated for 4 d. In step 3 (trans-differentiation), 100 nM glucagon-like peptide (GLP-1, Sigma-Aldrich, St. Louis, MO, USA) and 10 nM exendin-4 (Sigma-Aldrich, St. Louis, MO, USA) were added to the differentiation medium defined in step 2, and cells were incubated for 4 -day. Culture was performed for 10-day.

### 4.7. Measurement and Calculation of Islet Equivalents 

Trans-differentiated IPCs were subjected to Islet equivalent (IEQ) measurements. Whole cells in an uncoated 90 mm × 15 mm dish were photographed using an Olympus microscope (CKX53, Olympus, Tokyo, Japan) at 40× magnification and classified by size. The Olympus CellSens software (Olympus Corporation, Tokyo, Japan) was used to measure the diameter of all islets (µm) in the dish. The volume of each pancreas with an islet diameter of 150 μm was calculated by converting it into ‘1′ and then dividing it by fractions. Next, the IEQ was derived by multiplying the number of islets with a coefficient based on islet size (Table 1). The equation for calculating the coefficient is shown in Table 1, and the total number of islets as well as the total IEQ value were obtained using the described formulas [67,68].

### 4.8. Reverse Transcriptase-Polymerase Chain Reaction (RT-PCT)

IPCs were lysed in 600 μL of TRIzol reagent (Life Technologies, Gaithersburg, MD, USA). Subsequently, 200 μL of chloroform (Sigma-Aldrich, St. Louis, MO, USA) was added. The mixture was vortexed and then centrifuged (Eppendorf AG. Barkhausenweg 1. 22339 Hamburg. Germany) for 10 min at 13,000× *g* rpm. The supernatant was transferred to a new tube, mixed with isopropanol (Sigma-Aldrich, St. Louis, MO, USA) at a 1:1 (v:v) ratio, and centrifuged at 13,000× *g* rpm for 20 min. After washing with 70% ethanol (Sigma-Aldrich, St. Louis, MO, USA) and 100% ethanol (Sigma-Aldrich, St. Louis, MO, USA), the supernatant was removed from the tube, and the pellet was collected.

Each RT-PCR cycle included the following components: 14 μL of diethyl pyrocarbonate (DEPC, Sigma-Aldrich, St. Louis, MO, USA), 1 μg of RNA; 2.5 μL of 0.1 M DTT (Dithiothreitol, Invitrogen, Waltham, MA, USA), 5 μL of 5× First Strand Buffer (M-MLV RT Buffer, Invitrogen), 1.25 μL of dNTP (deoxynucleotides, Invitrogen, Waltham, MA, USA), 0.5 μL of Oligo (dT) 20 Primer (Invitrogen, Waltham, MA, USA), 0.25 μL (2000 units) of RNase inhibitor (Invitrogen, Waltham, MA, USA), and M-MLV reverse transcriptase (200 U/μL; Invitrogen, Waltham, MA, USA). The reactions were taking place at 37 °C for 1 h and at 72 °C for 10 min to synthesize cDNA. Subsequently, 2 μL of dNTP Mix (Takara, Tokyo, Japan), 0.2 μL of TaKaRa Taq (Takara, Tokyo, Japan), 1.5 μL of primer, and 10× PCR buffer (Mg^2+^) (Takara, Tokyo, Japan) were mixed with 4 μL of cDNA and then with 10.5 μL of DEPC water. The amplified samples were run on a 1% agarose gel, and images were captured using Q550CW image acquisition and analysis system (Leica Microsystems, Wetzlar, Germany). These were performed in five replicates. The primer pairs used for RT-PCR (BioRad, Hercules, CA, USA) are listed in Table 2.

### 4.9. Immunocytochemistry for Pancreatic Developmental Stage in IPCs

After trans-differentiating hUC-MSCs and hAD-MSCs into IPCs, the cells were fixed with 4% paraformaldehyde (PFA; Sigma-Aldrich, St. Louis, MO, USA), washed three times with 1× phosphate-buffered saline (PBS; Sigma-Aldrich, St. Louis, MO, USA), and incubated in a 0.2% PBS-containing Triton-X100 solution (PBST; (Sigma-Aldrich, St. Louis, MO, USA) for 20 min to permeabilize the cell surfaces. After three washes with 0.2% PBST, the cells were blocked in a solution of PBS containing 5% BSA (Sigma-Aldrich, St. Louis, MO, USA) for 2 h at 20–25 °C to block nonspecific staining. Next, 1:200 dilutions of primary antibodies specific for insulin (Cat#ab7842; Abcam, Cambridge, MA, USA), MAFA (Cat# ab98859; Abcam, Cambridge, MA, USA), and 1:200 dilutions of primary antibodies specific for C-peptide (Cat#ab14181; Abcam, Cambridge, MA, USA), in PBS with 5% BSA were added to the cells, which were then incubated at 4 °C overnight. The following day, cells were washed three times with 0.2% PBST and incubated at RT in a 1:1000 dilution of Alexa Fluor 488-conjugated guinea pig IgG (Cat# ab150185; Abcam, Cambridge, MA, USA), or Alexa Fluor 647-conjugated rabbit IgG (Cat# RSA1261; Bio-Rad, Hercules, CA, USA) secondary antibodies in PBS containing 5% BSA. After three washes in 0.2% PBST, the cells were finally incubated with a 1:20,000 dilution of Hoechst for 5 min and mounted using VECTASHIELD^®^ Mounting Medium containing DAPI (4′,6-diamidino-2-phenylindole, Vector Labs, Burlingame, CA, USA). Cells were then visualized under a fluorescence microscope (IX73, Olympus, TYO, JP) and transferred to a computer with a digital camera and the Olympus CellSens software for analysis.

### 4.10. Glucose-Stimulated Insulin Secretion Assay

For the Glucose-Stimulated Insulin Secretion (GSIS) assay, insulin secretion was measured by incubating 15 IPCs. First, IPCs were washed carefully with PBS and then incubated in Kreps Ringer Bicarbonate Buffer (Table 3; KRB buffer, Sigma-Aldrich, St. Louis, MO, USA), followed by incubation for 1 h in KRB buffer with 2.8 mM glucose (low glucose) or 17.5 mM glucose (high glucose). Insulin levels were measured in the supernatant after 1 h. Insulin secretion was determined using the human insulin ELISA Kit (Cat# ab278123; Abcam, Cambridge, MA, USA).

### 4.11. PKH26-Red Fluorescent Cell Linker

The hUC-MSCs for tracing of cells after transplantation were washed in supplement-free high-glucose alpha-MEM (Gibco Invitrogen Life Technologies, Grand Island, NY, USA) and centrifuged at 400× *g* for 5 min. Subsequently, cells were resuspended in 25 μL of culture solution and then mixed with 1 mL of Diluent C solution via pipetting. In another tube, 4 μL of a PKH26-Red solution (PKH26GL; Sigma-Aldrich, MO, USA) was added to 1 mL of diluent C solution and mixed. The PKH26-Red solution was subsequently added to the 1 mL cell suspension, incubated for 5 min, and 2 mL of FBS were added. After incubation for 1 min, the mixture was centrifuged at 400× *g* for 10 min to sediment the cells. The supernatant was discarded, and the cells were washed three times with 10 mL of complete culture medium and centrifuged for 5 min at 400× *g*. The PKH26-Red-stained hMSCs were placed in a 100 mm culture dish (Nunc, Naperville, IL, USA) and cultured for 3 days after PKH26-Red staining to induce their differentiation into IPCs.

### 4.12. Cell Viability 

Before transplantation, cells were incubated at 37 °C in a 95% air and 5% CO_2_ atmosphere for 24 h. After washing with PBS, MTT (3-(4,5-dimethylthiazol-2-yl)) solution (Thermo Fisher Scientific, Waltham, MA, USA) was added and the cells were incubated for 4 h. The culture medium was removed, and 150 μL of dimethyl sulfoxide (DMSO; Sigma-Aldrich, St. Louis, MO, USA) was added. Next, the sample was shaken for 10 min at 25 °C, and absorbance was measured at 540 nm with an ELISA reader (EPOCH, BioTek, Winooski, VA, USA).

### 4.13. Streptozotocin-Induced T1D Mouse Model 

**Figure 8:**
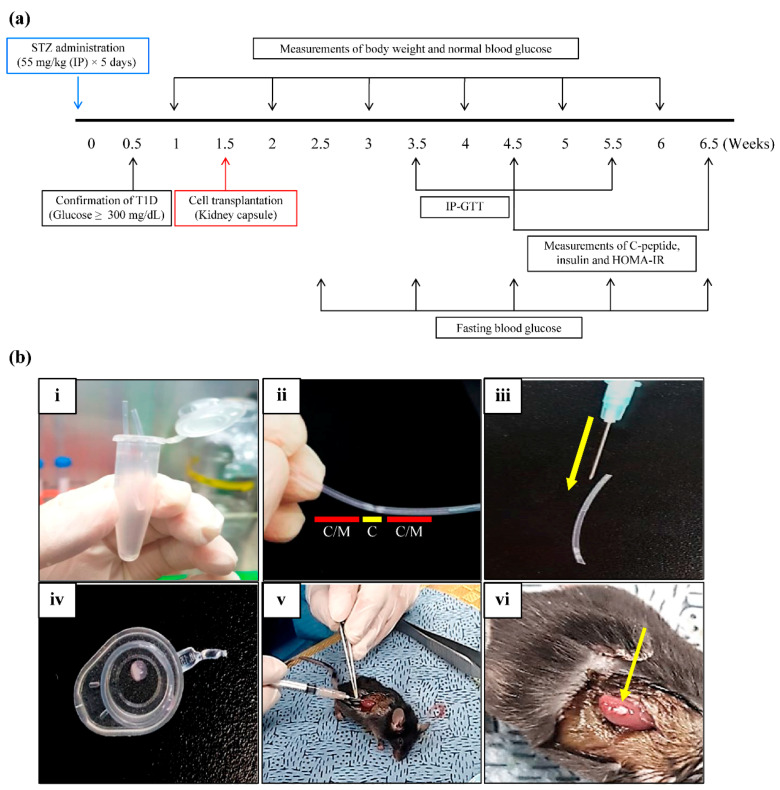
Establishment of the T1D mouse model, transplantation, and assessment. (**a**) After 3.5 and 5.5 weeks (2.5 and 4.5 weeks after cell transplantation, respectively), the animals were subjected to IP-GTT. At 4.5 and 6.5 weeks (3.5 and 5.5 weeks after cell transplantation, respectively), C-peptide and insulin levels were measured, and HOMA-IR was determined. Fasting blood glucose levels were measured weekly from 2.5 to 6.5 weeks. Body weight and normal blood glucose were measured weekly from week 1 to 6. At 2.5 and 6.5 weeks (3.5 and 5.5 weeks of cell transplantation, respectively), C-peptide and insulin levels were measured, and HOMA-IR was determined. At 6.5 weeks, the animals were sacrificed for analyses. (**b**) Cell suspension transplantation under kidney capsule. (**i**) Cells in culture medium were placed into a catheter, which was folded in half, placed into a 1.5 mL tube, and centrifuged. (**ii**) Cells in culture medium were assembled in the middle of the catheter, and the catheter was cut at one end to allow access to the cells. (**iii**) The catheter was carefully pushed forward using light pressure in a needle for cell transplantation (yellow arrow). (**iv**) When the syringe is pressurized, the cells with culture medium are transplanted stably. (**v**) The cells were implanted into the kidney capsule. (**vi**) Transplantation was performed successfully within the kidney capsule. Abbreviations: IP-GTT, intraperitoneal glucose tolerance test; HOMA-IR, homeostatic model assessment for insulin resistance; T1D, type 1 diabetes; STZ, streptozotocin. C/M, culture medium; C, cells.

A total of 40 C57BL/6 male mice (ORIENTBIO, SNM, KYG, KR) were included in the study. All experimental animal protocols used were approved by the Institutional Animal Care and Use Committees (IACUC) of EBO cooperation (approval number EBOA-2019-2), and all experimental procedures were performed in accordance with the institutional guidelines. Diabetes was induced by intraperitoneally injecting 12-h fasted mice with 55 mg/kg STZ in citric acid buffer at 4.5 pH (Sigma-Aldrich, St. Louis, MO, USA) for five consecutive days [69]. Mice were subsequently fasted for another 12 h, and Fasting Blood Glucose (FBG) levels were examined 72 h after STZ administration. Mice with induced T1D were selected using an FBG ≥300 mg/dL as a threshold [70]. For this experiment, mice were divided into the following four groups: G1, normal control; G2, T1D control, administered STZ (55 mg/kg body weight; Sigma-Aldrich, St. Louis, MO, USA) via intraperitoneal injection once a day (STZ dissolved in 0.1 mol/L citrate buffer, pH 4.5); G3, received STZ and were injected with hUC-MSCs (1 × 10^6^) one week after T1D induction; and G4, received STZ and were injected with differentiated hUC-MSCs into IPCs; hUC-IPCs (5000 IEQ) one week after T1D induction (Table 4). The in vivo experiments were designed according to the protocol depicted in Figure 8a.

### 4.14. Transplantation of hUC-MSCs and Induction of Cultured hUC-IPCs

Human UC-MSCs (1 × 10^6^ cells) or hUC-IPCs (5000 IEQs) were cultured, and mice in G3 as well as G4 were transplanted with one of the cell populations. All cells were prepared for transplantation via centrifugation at 2000× *g* rpm for 3 min. Following the removal of the supernatant (culture medium), the cells were resuspended in 40 μL of culture medium. Next, the cells in culture medium were placed into a catheter, which was folded in half, placed into a 1.5 mL tube (Cat# MCT-150-C, Corning, Axygen Scientific, Union City, CA, USA) and centrifuged at 12,000× *g* rpm for 5 min. The catheter was then cut at one end, and the cells collected in the middle of the catheter were then pushed forward with light pressure into a syringe for transplantation. Once a mouse was placed under respiratory anesthesia, the skin and fascia were incised, the kidneys exposed, and the cells were transplanted into the kidney capsule using a 26-gauge needle (Korea-vaccine, SEL, KR) (Figure 8b). The injection site was located at the upper or lateral side of the kidney to avoid damage to kidney blood vessels [71]. Upon completion of the cell transplantation, the transplantation site was cauterized, and the fascia as well as the skin were sutured to allow sufficient recovery. This method of transplanting hUC-MSCs or hUC-IPCs into the kidney capsule increased the transplantation rate and improved safety, ultimately decreasing mortality.

### 4.15. Evaluations of FBG, IP-GTT, C-peptide, Insulin, and HOMA-IR 

STZ-induced T1D was considered successful in animals exhibiting an FBG level ≥300 mg/dL (Figure 8a). FBG levels were measured weekly at the same time. Each mouse was subjected to IP-GTT after the administration of 2 mg of glucose/kg of body weight, and blood glucose levels in mouse tail vein samples collected at 15, 30, 60, and 120 min before glucose administration were measured with Glucotrend (Roche Diagnostics GmbH, Penzber, Germany) for 5 weeks. The evening before euthanasia, blood was collected from the abdominal vein of each mouse and centrifuged at 3000× *g* rpm for 15 min to separate the serum for blood biochemistry and insulin concentration measurements using ELISA. Quantitative estimations of serum insulin and C-peptide levels were performed using a mouse insulin ELISA kit (Cat# EZRMI-13K, Merck Millipore, Billerica, MA, USA) and a mouse C-peptide ELISA kit (Cat# 01-675-929, Invitrogen Life Technologies, Grand Island, NY, USA) 3 and 5 weeks after T1D induction. Insulin resistance was estimated using HOMA-IR and calculated using the following formula [72,73]:

HOMA-IR = fasting insulin (mU/L) × fasting glucose (mmol/L)/22.5 (in units/L)

### 4.16. Tissue Immunofluorescence Staining

Extracted tissues were perfused with 4% PFA and stored until a subsequent incubation in 30% sucrose for 24 h at 4 °C, followed by embedding in optimal cutting temperature (OCT) compound (Tissue-Tek; Sakura, Finetek, Torrance, CA, USA). The embedded samples were then stored at −80 °C and cut into 7–10 μm sections on a cryostat microtome (CM 1850; Leica). Subsequently, the frozen tissue sections were incubated at RT for 20 min and permeabilized for 20 min in PBS containing 0.2% Triton X-100. The tissue slides were blocked for 2 h with PBS containing 5% BSA (Sigma-Aldrich, St. Louis, MO, USA) and incubated at 4 °C overnight with an anti-insulin monoclonal antibody (Cat# ab7842; Abcam, Cambridge, MA, USA) with a 1:200 dilution. Following overnight incubation, cells were washed in PBS containing 0.2% Triton X-100 (3 times × 10 min). Cells were then incubated for 2 h with the respective secondary antibody at a 1:1000 dilution of Alexa Fluor 488-conjugated guinea pig IgG (Cat# ab150185; Abcam, Cambridge, MA, USA) or Alexa Fluor 647-conjugated rabbit IgG (Cat# RSA1261; BioActs, Kr) secondary antibodies in 5% BSA at 4 °C in a dark room. After washing, cells were mounted using VECTASHIELD^®^ Mounting Medium containing DAPI. Images were captured using a fluorescence microscope (Scope A1Carl Zeiss, Carl-Zeiss-Promenade 10, 07745 Jena, Germany), transferred to a computer with a digital camera, and analyzed using the ZEN software (Carl-Zeiss-Promenade 10, 07745 Jena, Germany). 

### 4.17. Statistical Analysis

Data were obtained from at least three to five independent experiments. The student’s *t*-test and one-way analysis of variance were used to determine significance. Statistical significance was set at *p* < 0.05.

## 5. Conclusions

Herein, we identified hUC-IPCs as candidate cells for T1D treatment based on their superior functional properties relative to those of hAD-IPCs. Blood glucose level reduction was confirmed in mice transplanted with hUC-IPCs compared to those transplanted with hUC-MSCs or the T1D control mice. Future studies should optimize the production of mature functional β-like cells in vitro, improve IPC differentiation efficiency from hUC-MSCs, and explore the protection of implanted IPCs from recipient immune attacks in the context of T1D treatment.

## Figures and Tables

**Table 1:** Equations for evaluation of Total IEQ and Total AI.

Islet Diameter Range (μm)	Islet Particle Number (AI)	IEQ Conversion Factor	IEQ Range
50–100		×0.167	Islet Diameter Range × AI × IEQ Conversion Factor
>100–150		×0.667	
>150–200		×1.685	
>200–250		×3.500	
>250–300		×6.315	
>300–350		×10.352	
>350		×15.833	
ΣAI		ΣIEQ	
Total AI = ΣAI × Dilution Factor			
Total IEQ = ΣIEQ (AI in each diameter) × Dilution Factor x			

Abbreviations: AI, actual islet; IEQ, islet equivalent.

**Table 2:** Primer Sets for RT-PCR.

Gene	Primer Sequence (Forward)	Primer Sequence (Reverse)	bp
*NGN3*	CGTGAACTCCTTGAACTGAGCAG	TGGCACTCCTGGGACAGATTTC	211
*NEUROD*	GAAAGCCCTCTGACTGAT	AAACTGGCGTGCCTCTAA	314
*INS*	CAGCCGCAGCCTTTGTGAAC	CAGGCTGCCTGCACCAGGG	170
*MAFA*	CTTCAGCAAGGAGGAGGTCATC	CTCGTATTTCTCCTTGTACAGGTCC	208
*GLUT2*	GCTACCGACAGCCTATTCTA	CAAGTCCCACTGACATGAAG	267
*SST*	CGTCAGTTTCTGCAGAAGTCC	CCATAGCCGGGTTTGAGTTA	196
*GAPDH*	AGCCACATCGCTCAGACACC	GTACTCAGCGGCCAGCATCG	303

**Table 3:** Composition of Krebs Ringer Bicarbonate Buffer including 0.2% bovine serum albumin.

Component	Final Concentration (mM)
NaCl	119
KCl	474
CaCl_2_	2.54
MgCl_2_	1.19
KH_2_PO_4_	1.19
NaHCO_3_	25
HEPES	10

**Table 4:** Experimental scheme of T1D induction in 8-week-old male C57BL/6 mice injected with STZ (55 mg/kg), hUC-MSCs (1 × 10^6^), or hUC-IPCs (5000 IEQ) intraperitoneally in G3 and G4, respectively.

Groups	
G1	Normal control
G2	T1D (STZ induction)
G3	T1D (STZ induction) with hUC-MSCs (1 × 10^6^)
G4	T1D (STZ induction) with hUC-IPCs (5000 IEQ)

Abbreviations: G, Group; STZ, streptozotocin; hUC-MSCs, mesenchymal stem cells derived from human umbilical cord; hUC-IPCs, insulin-producing cells derived from hUC-MSCs; T1D, type 1 diabetes; IEQ, islet equivalents.

## Data Availability

The data used to support our findings are included within the article.
